# Medical genetics studies at the SBB-2019 and MGNGS-2019 conferences

**DOI:** 10.1186/s12881-020-01109-8

**Published:** 2020-10-22

**Authors:** Ancha V. Baranova, Elena Yu. Leberfarb, Georgy S. Lebedev, Yuriy L. Orlov

**Affiliations:** 1 George Mason University, Fairfax, VA 22030 USA; 2 Research Centre for Medical Genetics, 115522 Moscow, Russia; 3 Institute of Cytology and Genetics SB RAS, 630090 Novosibirsk, Russia; 4 The Digital Health Institute, I.M.Sechenov First Moscow State Medical University (Sechenov University), 119991 Moscow, Russia; 5 Novosibirsk State University, 630090 Novosibirsk, Russia

Current medical genetics research is oriented on next-generation sequencing technologies, genomics studies [1, 2]. This special issue of BMC Medical Genetics presents the medical genetics works and case studies discussed at the “MGNGS-2019” conference in Suzdal, Russia in 2019, organized on the base of Moscow Medical Genetic Centre (http://ngs.med-gen.ru/). The Conference was focused on the discussion of the research on medical genetics and sequencing, and new omics technologies.

This journal issue contains materials on genetics and medical genetics presented in 2019 at the conference in Moscow and SBB-2019 (Systems Biology and Bioinformatics - 2019) School in Novosibirsk. The SBB School series on bioinformatics is organized annually since 2008 by the Institute of Cytology and Genetics of the Siberian Branch of the Russian Academy of Sciences and Novosibirsk State University (http://conf.bionet.nsc.ru/sbb2019/en/). We have published selected conference materials as special issues at BMC Genetics and related BioMed Central journals [3–5]. The SBB Schools in Novosibirsk are satellite events for BGRS\SB (Bioinformatics of Genome Regulation and Structure \ Systems Biology) multiconference [6], The special issue on medical genetics is accompanied by other BioMed Central post-conference journal issues in the genomics, bioinformatics, and medical genetics areas at BMC Bioinformatics, BMC Medical Genomics, BMC Genomics, and BMC Genetics, focusing on next-gen technologies applications [7–10]. We continued the BMC Med Genetics special issues in 2019 [1]. We believe such events and public discussion at the platforms of international publishers will bring the attention of the journal readers to actual medical challenges.

We open up this Special Issue by the population genomics study on schizophrenia by Evgeniya Poltavskaya et al. [11] (this issue). The schizophrenia has a large genetic component. It is characterized by a substantial genetic heterogeneity with contributions from common, rare, and de novo variants of a large number of genes, in addition to environmental factors [12]. The clinical heterogeneity of schizophrenia is expressed in the difference in the leading symptoms and course of the disease. *PIP5K2A* (Phosphatidylinositol-4-Phosphate 5-Kinase Type II Alpha) has been investigated as a potential susceptibility gene for schizophrenia.

The authors studied the possible association between eleven polymorphic variants of PIP5K2A and the clinical features of schizophrenia in a population of 384 white Siberian patients with schizophrenia using genotyping. *PIP5K2A* rs8341 and rs946961 showed significant association with course of schizophrenia (continuous or episodic). The experimental data confirm that *PIP5K2A* is a genetic factor influencing the type of course of schizophrenia in Siberian population. Disturbances in the phosphatidylinositol pathways may be a possible reason for the transition to a more severe continuous course of schizophrenia. The studies on medical genetics problems on Siberian populations were presented in [13, 14].

The work by Xianping Meng and colleagues [15] (this issue) continues association analysis topic using sequencing data. The authors discussed the molecular association between hyperkalemia and lung squamous cell carcinomas. Hyperkalemia is caused by abnormalities in the normal bone formation and degradation cycle, categorized by the calcium level in the blood serum. A strong association was reported between hyperkalemia and lung squamous cell carcinomas (LSCC) [16]. It is affecting patients in progressive stages, shortening survival times, and leading to poor prognosis. A mega-analysis using 11 independent LSCC RNA expression datasets was performed to test the hypothesis that genes influencing hyperkalemia may also play roles in LSCC. The results suggested that LSCC and hyperkalemia have a shared pathological basis and are mutual risk factors for each other. Genes associated with hyperkalemia may also play roles for the development of LSCC, and SPP1 could be a promoter for both hyperkalemia and LSCC.

Xixi Xiang and co-authors [17] (this issue) discuss therapeutic role of deep vein thrombosis inhibitor *SP1* in post-stroke patients. Deep vein thrombosis (DVT) has been well-known to be associated with stroke, which is also supported by many clinical studies [18]. To study the genes associated with DVT in the development of stroke performed literature-based disease-gene relationship data analysis, then conducted mega-analysis based RNA expression dataset mining. The results suggested that *SP1* could be a novel therapeutic target gene for post-stroke treatment.

Ludmila Savinkova et al. [19] (this issue) studied unannotated single nucleotide polymorphisms (SNP) in the TATA box of erythropoiesis genes and the associations with cognitive and mental disorders. There is growing evidence that different forms of anemia (changes in the number and quality of blood cells) may be involved in (or may accompany) the pathogenesis of various cognitive and mental disorders, such as Alzheimer’s and Parkinson’s diseases, depression of various severity levels, bipolar disorders, and schizophrenia. Higher hemoglobin concentrations in the blood may lead to hyperviscosity, hypovolemia, and lung diseases, which may cause brain hypoxia and anomalies of brain function, which may also result in cognitive deficits. In the study, a search for unannotated single-nucleotide polymorphisms of erythroid genes was performed using the authors software tool SNP-TATA_Z-tester, the Web service for in silico estimation of the affinity of TATA-binding protein (TBP) to a given SNP sequence [20, 21]. The uncovered candidate SNP markers of erythropoiesis anomalies may also be studied in cohorts of patients with mental disorders with comorbid erythropoiesis diseases. Applications of SNP marker analysis using TBP binding estimates in the gene promoters provide fundamental background for biomedical studies [22, 23].

Peter Sparber et al. [24] (this issue) present case report on myotonia congenita - a rare neuromuscular disease, which is characterized by a delay in muscle relaxation after evoked or voluntary contraction. Myotonia congenita can be inherited in a dominant (Thomsen disease) and recessive form (Becker disease) and both are caused by pathogenic variants in the *CLCN1* gene. To the publication date only one noncanonical splice site variant in the *CLCN1* gene was functionally characterized. The authors further contribute to this field by evaluation the molecular mechanism of splicing alteration in homozygous state reporting a clinical case of an affected patient.

Andrey Marakhonov and colleagues [25] (this issue) discuss prenatal diagnosis of Norrie disease. Hereditary ophthalmic pathology is a genetically heterogeneous group of diseases that occur either as an isolated eye disorder or as a symptom of hereditary syndromes [26]. The most challenging situation can arise when prenatal diagnosis of ophthalmic pathology is needed during an ongoing pregnancy. The case is for childbirth risk prognosis at 7–8 week of gestation because a previous child in the family, has congenital aniridia, glaucoma, severe psychomotor delay, and has had several ophthalmic surgeries. Considering the lack of pathogenic changes and precise diagnosis for the affected boy, NGS sequencing of clinically relevant genes was performed for the ongoing pregnancy; it revealed a novel hemizygous substitution in the NDP gene, which is associated with Norrie disease. Clinical polymorphism of hereditary ophthalmic pathology can severely complicate establishment of an exact diagnosis and make it time- and cost-consuming. NGS appears to be the method-of-choice in such complicated cases.

Overall, this journal issue includes reports of recent medical genetics applications, as well as case reports, continuing series on BMC Med Genetics and BMC Med Genomics special post-conference journal issues [1, 2]. We hope for continuing international exchange and education via the schools and competitions for young scientists (https://peerj.com/collections/72-bgrs-sb-2020/). We invite our readers worldwide to attend the systems biology meetings in Russia - Digital Medicine Forum (Digital Medicine Forum) and MGNGS-2020 (Medical Genetics - Next-Generation Sequencing) event postponed to 2021 (http://ngs.med-gen.ru/mgngs20/).
